# The Value of Mainstreaming Human Rights into Health Impact Assessment

**DOI:** 10.3390/ijerph111010076

**Published:** 2014-09-26

**Authors:** Gillian MacNaughton, Lisa Forman

**Affiliations:** 1 School for Global Inclusion and Social Development, University of Massachusetts Boston, 100 Morrissey Boulevard, Boston, MA 02125, USA; 2 Dalla Lana School of Public Health and Munk School of Global Affairs, University of Toronto, 155 College Street, Toronto, ON M5T 3M7, Canada; E-Mail: lisa.forman@utoronto.ca

**Keywords:** human rights, essential medicines, international trade agreements, intellectual property

## Abstract

Health impact assessment (HIA) is increasingly being used to predict the health and social impacts of domestic and global laws, policies and programs. In a comprehensive review of HIA practice in 2012, the authors indicated that, given the diverse range of HIA practice, there is an immediate need to reconsider the governing values and standards for HIA implementation [1]. This article responds to this call for governing values and standards for HIA. It proposes that international human rights standards be integrated into HIA to provide a universal value system backed up by international and domestic laws and mechanisms of accountability. The idea of mainstreaming human rights into HIA is illustrated with the example of impact assessments that have been carried out to predict the potential effects of intellectual property rights in international trade agreements on the availability and affordability of medicines. The article concludes by recommending international human rights standards as a legal and ethical framework for HIA that will enhance the universal values of nondiscrimination, participation, transparency and accountability and bring legitimacy and coherence to HIA practice as well.

## 1. Introduction

Health impact assessment (HIA) is increasingly used in a variety of settings to predict the health consequences of proposed domestic and global laws, policies and programs. There are many definitions of HIA, however, one widely cited definition was presented in the Gothenburg Consensus Paper:

Health impact assessment is a combination of procedures, methods and tools by which a policy, program or project may be judged as to its potential effects on the health of a population, and the distribution of those effects within the population [2].

Although there are many definitions, there is considerable consistency among scholars and practitioners in describing the basic elements of HIA [2,3,4]. First, it is generally accepted that HIA is prospective, in other words, it is carried out to predict future consequences and to inform decisions on interventions before they are undertaken rather than to evaluate impacts after a decision is made and implemented [4,5]. Second, there is widespread agreement on the procedural steps necessary to carry out HIA [2,4,6]. Third, participation by stakeholders, those potentially affected by the intervention, is also accepted as a core element of HIA practice. Indeed, more recent definitions of HIA tend to include participation of stakeholders as constitutive of HIA [4].

Despite consensus on these key features, there is no agreement on the values and standards governing HIA [3,7]. This challenge may reflect the distinct origins of HIA from three disciplinary communities, namely (1) environmental health, often associated with environmental impact assessment; (2) social determinants of health, focused on the underlying social causes of health conditions; and (3) health equity, focused on the differential impacts of interventions on specific populations groups [1]. While the environmental health perspective is directed toward *protection* of health or prevention of risks to health, the social determinants and health equity perspectives are aligned with health *promotion*. [3]. In addition to the distinct perspectives on the purposes of HIA, there is a disciplinary divide as environmental health HIA is based on epidemiology and toxicology, while health equity and the social determinants of health HIA are largely based in the social sciences with more qualitative data and an emphasis on participatory methods [3,5].

In view of the diversity of HIA practice, in 1999, the World Health Organization European Centre for Health Policy and the Nordic School of Public Health, in collaboration with the European Commission, held a consultation in Gothenburg to develop a common understanding of HIA. The resulting Gothenburg Consensus Paper outlines an approach to HIA, including the definition above, the key steps of HIA and importantly, the values governing HIA [2]. The six steps for HIA outlined in the consensus paper are: (1) screening for possible health impacts; (2) scoping of the health impact assessment; (3) assessment (via rapid health impact appraisal, health impact analysis or health impact review); (4) appraisal of the HIA report; (5) adjusting the proposed decision; and (6) monitoring and evaluation [2]. These are almost identical to those described in a major report by the US National Research Council on HIA published twelve years later in 2011 [4]. There is little disagreement on these steps, although they may vary in total number by combining or dividing some of the steps.

The Gothenburg Consensus Paper also set out a consensus among participants on five values governing HIA: (1) health—promoting the maximum health of the population; (2) democracy—meaning the right of people to participate in a transparent process for the formulation, implementation and evaluation of policies that affect their lives; (3) equity—emphasizing that HIA is concerned with the distribution of impact within a population in terms of gender, age, ethnic background and socio-economic status; (4) sustainable development—requiring that both short-term and long-term impacts are taken into consideration; and (5) ethical use of evidence—meaning that quantitative and qualitative evidence must be rigorous and incorporate different scientific disciplines to obtain as comprehensive an assessment as possible [2] (p. 4). The paper also acknowledges that “all policy processes are carried out in the framework of values, goals and objectives that may be more or less explicit in a given society and at a given time.” [2] (p. 4). The important point is that HIA should be explicit about its underlying values so that it contributes to a transparent decision making process in the specific context.

Since the Gothenburg Consensus Paper, HIA practice has grown considerably around the world in a great diversity of contexts and with differing perspectives. Not surprisingly, this diversity has continued to contribute to discussions—and controversy—over the underlying values of HIA [6]. For example, Kemm contended that HIA carried out with the underlying public health values of health, equity and participation was not an impartial assessment, and therefore would not fill the need that policy makers have for impartial advice [5]. Scott-Samuel and O’Keefe disagreed, maintaining that the application of values in HIA does not equate with partiality or bias in the assessment [7]. More recently, Povall *et al.* recommended that values—including decisions on equity—be negotiated and made explicit during the scoping phase of HIA [6] (p. 9). In a comprehensive review of HIA practice in 2012, Harris-Roxas *et al.* indicated that “there is a pressing need to revisit, at an international level, the governing values and standards that apply to HIA’s use in order to ensure they are relevant to the current diverse range of HIA practice” [1] (p. 49). 

Independent from the evolution of HIA, about twenty years ago human rights scholars began to develop and test methodologies for human rights impact assessment as a tool for governments to ensure that their policies are consistent with their legal obligations to respect, protect and fulfill human rights. Notably, some human rights scholars also recommended integrating human rights standards into the other forms of impact assessments [8]. In light of the debate on HIA values, scholars and practitioners in both human rights and health impact assessment communities have suggested that integrating human rights into health impact assessment could benefit both fields [7,9]. In particular, human rights may provide an ethical and legal framework for HIA, while HIA may provide a rigorous evidence-based method of assessment for achieving health and human rights goals. Drawing on these recommendations, this article proposes that international human rights standards be integrated into HIA to provide a universal system of values and standards for HIA that is also backed up by international and domestic laws and mechanisms of accountability.

Following this introduction, Section 2 defines the sources and content of international human rights laws, principles and standards and explains how human rights laws, principles and standards could be integrated into HIA. Section 3 illustrates the idea of mainstreaming human rights into HIA with examples of impact assessments that have been carried out to predict the potential impacts of intellectual property rights in international trade agreements on the availability and affordability of medicines. Section 4 considers a range of potential benefits from integrating human rights into HIA. The article concludes in Section 5 that international human rights standards provide a legitimate ethical and legal framework for HIA that has an established broad consensus around the world.

## 2. Human Rights and Impact Assessment

### 2.1. International Human Rights Law, Principles and Standards

The modern international human rights framework is built on the Charter of the United Nations, which establishes that one of the main purposes of the organization is to achieve international cooperation “in promoting and encouraging respect for human rights and for fundamental freedoms for all without distinction as to race, sex, language or religion” [10] (article 1). All 193 members of the UN have pledged to promote universal respect for, and observance of, human rights [10] (article 1). These rights are further detailed in the Universal Declaration of Human Rights, which was adopted by the UN General Assembly in 1948 [11]. The Universal Declaration is the most widely known pronouncement of fundamental human rights around the world. Subsequently, human rights were codified in a several international human rights treaties imposing legal obligations on the countries that ratify them.

There are several essential features of human rights as they are set out in these international instruments. First, human rights are universal, meaning that all people everywhere are entitled to human rights [12]. Second, international human rights law provides that all people are born equal in dignity and rights and that they are entitled to all rights without discrimination on the basis of race, color, sex, language, religion, political or other opinion, national or social origin, property, birth or other status [11] (articles 1,2). Third, people are entitled to take part in public affairs and to have meaningful input into the public decisions that affect their lives [11,13]. Fourth, people are entitled to hold their governments accountable for ensuring their human rights and to an effective remedy when these rights are violated [11,13]. Finally, all human rights are inter-related and interdependent, meaning that the realization of one right, such as the right to education, increases the opportunities for exercising more fully other rights, such as the rights to health and to work [12]. In sum, universality, equality and nondiscrimination, participation, accountability and interdependency are overarching human rights principles, based in international human rights law, that provide a legitimate and coherent basis for policy and other decision making.

In addition to these overarching principles—based in international human rights law—the human right that is most relevant to HIA is the right to the highest attainable standard of mental and physical health, also known as the “right to health”. In 1946, the Constitution of the World Health Organization was the first international instrument to declare that “[t]he enjoyment of the highest attainable standard of health is one of the fundamental rights of every human being without distinction of race, religion, political belief, economic or social condition” [14]. In 1948, the Universal Declaration of Human Rights recognized that “everyone has the right to a standard of living adequate for the health and well-being of himself and of his family, including food, clothing, housing and medical care and necessary social services” [11] (article 25). Notably, all members of the UN are required to go before the UN Human Rights Council every four years to report upon their progress in implementing their international human rights obligations, including those rights enshrined in the Universal Declaration.

The right to the highest attainable health has also been codified in several international human rights treaties, including the International Covenant on Economic, Social and Cultural Rights (ICESCR) and the Convention on the Rights of the Child, that impose legal obligations upon the countries that ratify—in other words—sign and assent to—them [15,16]. All but two countries in the world—the United States and Somalia—have ratified the Convention on the Rights of the Child, and 166 countries have ratified the ICESCR. In essence, the right to health is a universally recognized human right. The most detailed provision on the right to health is in the ICESCR. It provides that all parties to the Covenant shall take steps to achieve the full realization of the right to health by, among other means, reducing infant mortality, improving environmental and occupational health, preventing and treating epidemics and occupational diseases, and ensuring health care services to all [15] (article 12).

In 2000, the Committee on Economic, Social and Cultural Rights, the UN body responsible for monitoring implementation of the ICESCR, issued a general comment providing further details on the right to health. The comment clarifies that the right to health is not a right to be healthy, but rather a right to a health system—including health care and the underlying determinants of health—that provides the opportunity for people to attain the highest possible standard of health [17] (paragraph 8). Like other complex rights, the right to health encompasses a bundle of procedural and substantive rights. For example, the right to a fair trial includes the right to an attorney, the right to cross-examine witnesses who testify against you and the right to remain silent. It does not guarantee a verdict of “not guilty” but rather is intended to make the outcome of the trial fairer. Similarly, the right to health encompasses a number of rights—like the rights to nutritious food and potable water, the right to essential medicines, the right to health information and the right to participate in health policymaking, among others—that all contribute to making health outcomes fairer. Importantly, the general comment provides that the government has the obligation to ensure that health care and the underlying determinants of health are available, accessible, acceptable and of good quality [17] (paragraph 12).

*Availability* means that public health and health care facilities, goods and services must be available in sufficient quantity in the country. *Accessibility* has four dimensions. It means that health care and the underlying determinants of health must be (1) accessible to all on a nondiscriminatory basis; (2) physically accessible and within safe physical reach of all sections of the population; and (3) economically accessible and affordable for all. Additionally, health information must be accessible to all, and people have a right to seek, receive and impart health information. *Acceptability* means that health care and the underlying determinants of health must be respectful of medical ethics, culturally appropriate and gender sensitive. *Quality* means that health care and the underlying determinants of health must be scientifically and medically appropriate and of good quality, including, for example, skilled health care personnel, scientifically approved drugs and hospital equipment, safe water and adequate sanitation [17] (paragraph 12).

The 166 countries that are parties to the ICESCR must report to the Committee on Economic, Social and Cultural Rights on a regular basis on their implementation of the Covenant, including their progress in ensuring the full realization of the right to the highest attainable standard of health. Moreover, under a new procedure established in 2013, people may bring complaints to the Committee when they believe that their rights, including the right to health, have been violated. Similarly, there is a Committee on the Rights of the Child that oversees implementation of the Convention on the Rights of the Child and the child’s right to the highest attainable standard of health and requires all 193 parties to report regularly on their progress in doing so.

The human rights principles of universality, equality and nondiscrimination, participation, accountability and interdependency, together with the right to health framework of availability, accessibility, acceptability and quality, provide a human rights framework for assessment of policy, program and project proposals. This framework is widely accepted around the world and entrenched in international human rights law with multiple international and domestic mechanisms of accountability, including the UN Human Rights Council, the human rights committees and domestic courts. As almost all countries are bound by these laws, principles and standards, they provide a legitimate ethical and legal framework for health impact assessment.

### 2.2. Human Rights Impact Assessment

While HIA has developed over the past three decades in three areas of public health—environmental health, social determinants of health and health equity—human rights impact assessment (HRIA) has evolved along a separate path largely disconnected from these public health efforts. At the international level, as early as 1979, the Secretary General of the United Nations recommended considering “the practicability of requiring a ‘human rights impact statement’, which might be similar in concept to an environmental impact statement, to be undertaken prior to the commencement of specific development projects or in connexion with the preparation of an overall development plan or programme” [18] (p. 160). It was not until 1994, however, that public health and human rights impact assessment were linked by Lawrence Gostin and Jonathan Mann in an article describing a methodology for assessing the human rights effects of public health policies [19]. In their article, public heath policies were viewed as potentially at odds with civil and political human rights. At that time, the importance of civil and political rights was widely acknowledged by health advocates, however, economic and social rights were comparatively little known [20].

Since Gostin and Mann’s pioneering article, HRIA has evolved considerably. One reason for the great changes in health-related HRIA is that the right to health has been defined in much more detail since 1994. While the right to health encompasses traditional civil and political rights, like the rights to confidentiality and the right to participate in health policy decision-making, it also has economic, social and cultural aspects, such as the right to health care without great financial risks and the right to respect for diverse cultures in health care delivery. Notably, the right to health includes both public health and heath care elements. In addition to the continued “unpacking” of the right to health, health-related HRIA has also developed considerably since 1994 because nongovernmental organizations (NGOs) have taken up this method to hold governments accountable for the human rights impacts of their decisions.

For example, a Canadian NGO called Rights and Democracy developed one of the first HRIA methodologies in 2004 to assess human rights impacts of foreign direct investment projects. Its methodology integrates a human rights-based approach by assuring transparency, accountability and nondiscrimination, focusing on vulnerable groups and recognizing the indivisibility of human rights [21]. One of the most prominent examples of HRIA in the health context is the Health Rights of Women Assessment Instrument (HeRWAI) developed by a group of NGOs in the Netherlands, Kenya, Malaysia, Nicaragua and Bangladesh as an advocacy tool to bolster international mechanisms for monitoring state accountability for women’s rights [22]. The HeRWAI tool uses human rights standards from the ICESCR and the Convention of the Eliminations of Discrimination Against Women within a step-wise HIA approach to identify how women’s rights are affected, elaborate government commitments and develop recommendations to enhance women’s health rights [23]. NGOs around the world have used HeRWAI as the basis for their advocacy efforts [22]. 

Stand-alone HRIA methodologies of this type are particularly well suited to human rights NGOs, human rights commissions and others that are unlikely to carry out other types of impact assessment [24,25]. Additionally, human rights scholars and practitioners have recommended that governments in particular, integrate human rights into the other forms of impact assessment, including HIA [9,24,25,26]. Public health scholars have also given specific attention to the links between health and human rights, as well as between HIA and HRIA, recognizing such tools as capable of predicting the health and human rights consequences of proposed public policies in a range of domains including international relations and foreign policy [7,20,27].

### 2.3. Integrating Human Rights into Health Impact Assessment (HIA)

The idea of integrating human rights into other forms of impact assessment, in particular HIA, was raised by public health scholars O’Keefe and Scott-Samuel over a decade ago and a few years later again by Paul Hunt, then UN Special Rapporteur on the right to health [20,28]. The so-called “mainstreaming approach” offers a number of advantages over stand-alone HRIA. First, as HRIA is a comparatively new methodology, by integrating human rights into HIA, human rights practitioners could take advantage of HIAs more established expertise and methodologies [24,25].

Additionally, governments or other entities that are already carrying out HIAs are not likely to want to take on the further burden of carrying out another type of impact assessment. In such cases, it may make more sense to integrate human rights into HIA or the other types of impact assessment already being carried out. [8,24]. Third, the mainstreaming approach, when carried out by governments, is also an appropriate means for meeting their international human rights obligations to ensure that proposed policies, programs and project do not have negative effects on the right to health, but rather further the government’s duty to respect, protect and fulfill all human rights.

Finally, by mainstreaming human rights into HIA, the process serves to educate policy makers about their human rights obligations and also helps to “focus policy making on the wellbeing of people, especially vulnerable groups” [25]. In this respect, mainstreaming human rights into HIA is highly suitable for governments to comply with their obligations to progressively realize the right to health, while the stand-alone HRIA is well suited to human rights commissions and human rights NGOs lobbying for governments to comply with this right [25].

Hunt and MacNaughton presented seven principles for integrating human rights into HIA or other forms of impact assessment and considerations for taking a human rights approach at each step of the HIA process. The seven human rights principles are: (1) use an explicit human rights framework; (2) aim for progressive realization of rights; (3) promote equality and nondiscrimination in the policy process; (4) ensure meaningful participation of all stakeholders; (5) provide information and protect the right to free expression; (6) establish accountability mechanisms for the state; and (7) recognize the interdependence of all human rights [8]. These principles align closely with the UN Common Understanding of a Human Rights-Based Approach to Development Cooperation [12].

Similarly, Walker explicitly draws on the UN Common Understanding and suggests that a human rights-based approach to impact assessment should comprise four key elements [29]. First, human rights should be the explicit goals of the impact assessment. In this respect, the primary goal of a human rights-based HIA would be to progressively realize the right to health. Second, the process of the impact assessment should respect human rights, including the principles of equality and nondiscrimination, participation and accountability. These principles parallel the Gothenburg Consensus values of democracy (participation) and equity (equality and non-discrimination), but notably also include the dimension of accountability. From the perspective of HIA, the Gothenburg consensus importantly adds sustainable development (consideration of short-term and long-term impacts) and ethical use of evidence (rigorous use of different scientific disciplines and methodologies). Third, the impact assessment should contribute to developing the capacities of “duty-bearers” to meet their obligation and “rights-holders” to claim their rights. This education function in HRIA—also present in HIA—is distinctly grounded in human rights and is closely linked to empowering communities. Finally, the impact assessment should involve international and domestic human rights mechanisms and actors [29], which will also educate duty-bearers and rights-holders about these mechanisms of accountability.

In terms of integrating human rights into the step-wise process of HIA, there are factors to consider at each step. Previous publications provide detailed lists of human rights considerations for each step of the impact assessment [8,21,29]. Here we present only a few of these considerations to illustrate what a human rights framework brings to the process. First, at the screening step, it is important to examine the government’s legal obligations for the right to health in determining whether to recommend carrying out a full HIA of a proposed policy or program as the conclusion could be made in terms of specific legal obligations for the right to health. Second, at the scoping stage, affected populations and vulnerable groups should be educated about their right to health and their right to participate in the HIA. In addition, the research questions and the analysis plan should be based on an explicit human rights framework, and the team selected to carry out the impact assessment should include expertise in human rights. Third, at the data collection stage, human rights calls for participation of marginalized and disadvantaged groups and attention to their concerns. Fourth, in the analysis or assessment stage, the evidence collected must be compared to, and analyzed on the basis of, the obligations that the government has for the right to health that were determined at step 1. Recommendations with respect to the available options should be made on the basis of their potential for contributing to the full realization of the right to health. At the fifth stage, the report must make its recommendation on the basis of a human rights rationale; in other words, it should spell out the best options for the government to take to fulfill it human rights obligations. On the basis of this rationale, the stakeholders may hold their government accountable for the decision that it makes. Finally, at the sixth stage, the monitoring and evaluation plan must include the means in which stakeholders may participate in continuing to improve the policy or program and its implementation as well as the mechanisms for stakeholders to bring complaints for concerns about impacts of the policy or project on their right to health. Overall, each step of the HIA may be tailored to a human rights framework embedding the underlying human rights value of human dignity and the human rights principles of universality, equality and nondiscrimination, participation, accountability and interdependency in the assessment process and the recommendations.

## 3. Human Rights-Based Impact Assessments of International Trade Agreements

The need for human rights-based HIA has come to greatest prominence in relation to the potential impact of international trade agreements on access to medicines in low- and middle-income countries. Concerns that these agreements threaten the realization of human rights, particularly the right to health, have prompted numerous human rights bodies to call upon governments to conduct assessments of international trade rules to ensure that proposed laws, policies or programs are consistent with government human rights obligations, particularly the right to heath, under international and domestic law [30,31,32,33,34,35,36]. In particular, Hunt, then United Nations Special Rapporteur on the Right to Health, recommended that urgent attention be given to developing a methodology for right to health-specific impact assessments of trade rules [37].

In response to these concerns, NGOs and scholars have increasingly used varying impact assessment methodologies in relation to international trade agreements to predict the effects that proposed intellectual property provisions would have on the availability and affordability of essential medicines. Many of these impact assessments have not used an explicit human rights framework [38,39,40,41,42]. On the other hand, several assessments have specifically engaged human rights [29,43,44]. Walker developed the most detailed and rigorous HRIA methodology of trade agreements to date, making a significant contribution to HRIA methodology that is specifically adapted to the idiosyncrasies of the trade context [29,45]. He applied his methodology to assess the impact of a prospective free trade agreement between the U.S. and Costa Rica on access to medicines in Costa Rica [29,45].

Despite the fact that governments hold primary responsibility for realizing human rights, only one HRIA of trade-related intellectual property rights has been conducted at the behest of a low- or middle-income government. In 2006, the Thai National Human Rights Commission conducted an assessment of the human rights impacts of a free trade agreement being negotiated with the United States in a range of sectors including health [42,43]. Although the Commission did not integrate an explicit human rights framework into its methodology, leaving much to be improved upon in the future exercise of HRIA by governments, it was pioneering in using impact assessment to predict impacts of proposed trade rules on health and human rights.

The imperative for HRIA of trade agreements and the specific challenges in this domain prompted successive expert consultations in 2010 and 2011, which proposed guiding principles and methodologies [46,47]. Importantly, in 2011, De Schutter, then UN Special Rapporteur on the right to food, issued *Guiding Principles on Human Rights Impact Assessments of Trade and Investment Agreements* [47]. The *Guiding Principles* indicate that States are bound by their pre-existing international treaty obligations, such as their pre-existing human rights obligations, and therefore they are prohibited from entering into new international treaties that would impose inconsistent obligations. Human rights impact assessment is a tool to ensure consistency and coherence between the pre-existing legal obligations of the State and proposals put forth in international trade negotiations. In this respect, De Schutter suggests that human rights impact assessment in the context of international trade negotiation is not simply good policy but a human rights legal obligation [47] (p.5). As a result, the *Guiding Principles* recommend that all States “prepare human rights impact assessment prior to the conclusion of trade and investment agreements” [47] (p.5).

Although few countries have adopted legal requirements to carry out HIA [4], there is an emerging view that human rights impose a legal requirement upon governments to carry out HRIA or human rights-based HIA in the context of trade negotiations where proposed intellectual property provisions may affect health and human rights [37,47]. Importantly, HRIA and human rights-based HIA may help to ensure that governments do not enter international agreements that conflict with their pre-existing obligations for human rights [47] (p.5). This rationale has wider applicability as well. Indeed, governments should carry out human rights-based HIA in a multitude of contexts to ensure that proposed policies, programs and projects do not conflict with their pre-existing legal obligations to progressively realize the right to the highest attainable standard to health.

## 4. The Potential Value of a Human Rights Framework for Health Impact Assessment (HIA)

Responding to the 2012 call to revisit the values and standards that are important to HIA practice, we propose that human rights in general, and the right to health in particular, may provide a useful ethical and legal framework for HIA in diverse contexts. Indeed, integrating human rights into HIA could provide multiple advantages. First, human rights provide a set of governing values, standards and goals for HIA and root the methodology within the complementary project of advancing wellbeing inherent to the project of human rights. Moreover, human rights is a legitimate framework of values and standards to adopt for HIA because they are a widely accepted set of values, and governments around the world have voluntarily agreed to abide by these rights. People, therefore, have a legitimate expectation that governments will make efforts to implement the right to health and other human rights [25].

Second, human rights establish a legal obligation—at least in certain circumstances, such as international trade negotiations—to carry out human rights-based HIA. Over time, it is likely that the legal obligations to carry out human rights-based impact assessments will expand even if only at first to ensure that States are not putting human rights at risk, or adopting conflicting laws and policies, by adopting proposed policies, programs and projects. Ideally, the obligation to carry out HIA would be explicitly entrenched in law [4] (pp. 56,56), and entrenching HIA practice in a human rights legal framework brings it one step closer to such entrenchment. Already, using a human rights framework will link HIA to established mechanisms of accountability at both the domestic and international level, empowering people to hold their governments accountable to carry out the HIA and to follow the recommendations. This is perhaps the most important contribution that human rights brings to HIA, the insistence that government policy making and implementation be accountable to the people it affects.

Third, human rights is a framework that applies to all levels and all sectors of government, and therefore it promotes coherence in government policy making and functioning to use this framework to develop goals and govern practices across all divisions of government [8]. In the case of international trade negotiations, for example, the human rights framework applies equally to the department of commerce and trade as it does to the department of health, and thus these sectors must work toward the same goals of improving individual wellbeing and realizing the right to health. Human rights makes clear that the government and the economy must work to achieve people-focused goals, rather than people working to achieve government’s goals for the economy.

Fourth, the principles of participation, equality and nondiscrimination (health equity), and access to information, all valued practices of HIA, are also ethical and legal obligations imposed by human rights [8]. Thus, ensuring that HIA follows these principles is not just good practice, it is grounded in the international human rights framework beginning with article 1 of the Charter of the United Nations, which prohibits discrimination on the basis of race, sex, religion or language and commits all UN members to promote universal respect for human rights [10].

Fifth, today the right to health has been well developed by the international human rights mechanisms and domestic courts as well. This body of jurisprudence provides a wealth of authority for human rights and HIA practitioners to use to support the need for human rights-based HIA, the goals of HIA and the principles underlying the process of HIA. As these bodies—courts, human rights treaty bodies and UN Special Rapporteurs—are legitimately authorized to determine the content of the right to health—their work can also be useful to people in the field advocating for the recommendations of an HIA. Moreover, the right to health features, such as availability, accessibility, acceptability and quality, provide the basis for policy analysis, advocacy and mobilization.

Finally, the human rights framework is complementary and compatible with HIA. The idea that all human rights are interdependent is consistent with the public health notion of the social determinants of health, which links to numerous rights—the rights to water, housing, food, health, education, a clean environment, safe working condition—in much the same manner. Indeed, both human rights and HIA aim to achieve the wellbeing of individuals and communities. While there is substantial overlap and compatibility, human rights and HIA can also complement each other. Human rights provide an ethical and legal framework, while HIA provides a rigorous evidence-based methodology to guide decision making.

## 5. Conclusions

Human rights cannot always provide an easy answer to specific policy questions. Nonetheless we believe that it provides a highly legitimate value-based framework for decision making at the local, national and global level. Human rights therefore may provide a basis for resolving the lack of consensus on the underlying values and standards of HIA. Whether HIA is conducted as a stand-alone assessment or as part of an environmental impact assessment or any other type of impact assessment, the same human rights laws, principles and standards apply. Governments have the obligations to respect, protect and fulfill the right to the highest attainable standard of physical and mental health and to ensure that people are able to participate in a transparent, nondiscriminatory decision-making process on issues that affect their lives. Human rights provide the means to hold governments accountable for ensuring that these values underlie HIA practice.
